# The value of general health perception in health equity research: A community-based cohort study of long-term mortality risk (Finnmark cohort study 1987–2017)

**DOI:** 10.1016/j.ssmph.2021.100848

**Published:** 2021-06-18

**Authors:** Knut Fylkesnes, Monika Dybdahl Jakobsen, Nils Oddbjørn Henriksen

**Affiliations:** a Centre for International Health**,** University of Bergen, Norway; b Centre for Care Research North, Department of Health and Care Sciences, UiT The Arctic University of Norway, Tromsø, Norway; c Department of Health and Care Sciences, UiT The Arctic University of Norway, Tromsø, Norway

**Keywords:** Mortality, Cohort study, Health equity, Self-rated health, Health status, Work disability pension, Norway

## Abstract

**Background:**

General health perception as measured by self-rated health (SRH) is an individual's synthesis of personal overall health and has value in its own right. In addition, this subjective perspective has a unique predictive power of subsequent mortality and adds valuable information not captured by objective measures. We studied the relationship between SRH and subsequent mortality to demonstrate how simple self-ratings can enhance our understanding of health inequities.

**Methods:**

Data from a population-based survey conducted in Finnmark 1987/1988 were linked to the Norwegian Cause of Death Registry for information on all deaths by the end of 2017. We used Cox proportional hazard regression modelling to estimate the relative effects of all-cause mortality separately for sex and age (30–49 and 50–62 years) with stepwise adjustment for socio-demographics and various other health status and behavioural measures.

**Results:**

The age-adjusted power of mortality prediction of SRH was strong (most pronounced in the youngest age-group) but markedly attenuated by other factors. Education inequality in mortality was most substantial in the youngest age-group, which might partly be due to a combination of selective mortality and historical changes in health inequality. In comparison, educational inequality in SRH was clearly pronounced regardless of age. Work disability pension appeared as the common key factor affecting the mortality prediction of SRH and educational inequity for both subsequent mortality and SRH.

**Conclusion:**

SRH adds unique information to our understanding of health inequities. The consistency in shared predictors of educational inequity concerning both mortality and SRH underscores the correspondence of these measures. In addition to predicting the fatal effects of social selection mechanisms, SRH adds non-fatal effects and seems less prone to selective mortality. The results are relevant to approaches in health equity research and have important policy implications.

## Highlights

• Self-rated health yielded robust age adjusted mortality predictions.
• Self-rated health adds unique information to our understanding of health inequities.
• Educational inequity in mortality is extensive, but it narrows with age, which can be explained by selective mortality and historical changes.
• Measures to reduce preventable workforce exclusions can be effective for achieving or maintaining health equity.

## 1 Introduction

General health perception or self-rated health (SRH) can be considered an individual's synthesis of both subjective and more objective information about their health. Thus, SRH provide unique information not captured by what can be measured independently of individual experiences (the objective perspective).

An extensive amount of previous research has shown that the simple question “How would you evaluate your health overall?” has a strong predictive ability concerning different future life events and behaviours, and in particular has the unique power to predict mortality (DeSalvo et al., 2006; Idler & Benyamini, 1997; Jylha, 2009). This predictive power is consistent among adults in most world regions (Jylha, 2009; Lorem et al., 2020; Vie et al., 2019). For example, SRH was a substantial predictor of both mortality and HIV infection in an African cohort experiencing extremely high HIV prevalence and related mortality, even though few individuals were tested (Dzekedzeke et al., 2008; Siziya & Fylkesnes, 2005). High predictability of SRH has also been documented for a variety of other outcomes. This includes subsequent health care use (DeSalvo et al., 2005; Miilunpalo et al., 1997) and prescribed medication in adulthood (Hetlevik et al., 2019; Vie et al., 2018). It is also the key variable associated with both patient-initiated and provider-initiated health care use (Fylkesnes, 1991, 1993; Fylkesnes & Forde, 1991; Fylkesnes et al., 1992). Finally, SRH has been reported as a useful tool for clinical practices (Unden & Elofsson, 2001; Waller et al., 2015).

The strong mortality prediction of SRH has led to its widespread use, and its use in health equity assessments is a case in point (de Bruin et al., 1996; Kunst et al., 2005). Equity in health is an ethical concept defined as “the absence of systematic disparities in health between social groups who have different levels of underlying social advantage/disadvantage” (Braveman & Gruskin, 2003; Marmot et al., 2008; Whitehead, 1992). The WHO Commission on Social Determinants in Health stated that health and illness follow a social gradient: the lower the socioeconomic position (Galobardes et al., 2007), the worse the health (Marmot et al., 2008). Educational attainment is considered one of several interrelated indicators of socioeconomic position, and has been demonstrated to capture differences in socioeconomic circumstances that influence health and mortality (Galobardes et al., 2007).

One rationale for studying SRH for use in health equity research has often been that its validity with reference to mortality needs to be tested, and testing this relationship is often referred to as “true health” (Dowd & Zajacova, 2007; Huisman et al., 2007). The question has been to what extent the predictive power of SRH for mortality correlated with indicators of socioeconomic position. Cohort studies from different countries have given mixed answers to this question (Burstrom & Fredlund, 2001; Dalen et al., 2012; Dowd & Zajacova, 2007; Holseter et al., 2015; Huisman et al., 2007; McFadden et al., 2009; Regidor et al., 2010; Singh-Manoux et al., 2007; van Doorslaer & Gerdtham, 2003). Given that SRH predicts mortality equally well for high and low socioeconomic positions, the interpretation has been that this measure will function well as an indicator for use in health-equity studies. For instance, if mortality predictions are more accurate for a high versus low socioeconomic positions, social inequality would be likely to be overestimated. However, this rationale of testing the validity of SRH against mortality underrates the perspective of the individual in health equity assessments. Similarly, since mortality is not likely to comprise non-fatal illness, injury or accident, inequalities in SRH cannot just be generalised to the expected inequality level in mortality.

To address the accuracy of SRH and enhance our understanding of the value of SRH for use in health equity studies, we analysed mortality risk among adults in a 30-year follow-up cohort study to investigate the relationship between SRH and mortality. We have previously used this base-line data in this cohort study to analyse factors and dimensions involved in SRH (Fylkesnes & Forde, 1992). The present investigation had four major objectives: first, to examine the mortality prediction of SRH; second, to measure if or to what extent the mortality prediction of SRH differs by educational attainment; third, to examine the education inequality in and determinants of mortality; finally, to examine education inequalities in SRH as compared to the respective education inequality in mortality.

## 2 Methods

### 2.1 Materials

The third Finnmark County Health Survey (Bjartveit et al., 1979) conducted by the National Health Screening Services from March 1987 to June 1988 formed the base-line for this cohort study. All county residents aged 40–62 years and a representative 20% sample of all residents 30–39 were invited. The survey included a personal examination (physiological measurement including cholesterol level, blood pressure, serum lipids, and body-mass index) and three self-administered questionnaires (the first issued as part of the invitation, the second presented at the examination to be returned by surface mail, and the third issued to all invited persons by surface mail three weeks after the examination). The questionnaires were in the Norwegian and Sami languages and contained a variety of questions including socio-demographics, work-related aspects, dimensions of health status, indicators of lifestyle/behavioural aspects and diet.

A total of 8928 men (84% of all invited, mean age 48.00) and 8603 women (91% of all invited, mean age 47.97) turned up for the first interview and for the personal examination. All examined participants responded to the first questionnaire, whereas 72% of them responded to all questionnaires. The analyses performed in this paper were based on information from both the personal examination and all the questionnaires. Selection bias due to non-response to questionnaires has been previously studied based on these baseline data (Fylkesnes & Forde, 1992). The possibility of distortion of associations due to non-response to questionnaires could be studied by including and excluding non-responders, respectively. These distortions were not found to be of sufficient magnitude to substantially bias estimates (Fylkesnes, 1991; Fylkesnes & Forde, 1992).

Participants in the baseline survey were identified with dates and causes of death by linkage to the Norwegian Cause of Death Registry (Bakken et al., 2015), i.e., during the 30 years since the survey examination (covering all deaths until end of 2017). The Norwegian Institute of Public Health was instrumental in providing the data.

### 2.2 The analytical model and variables

We previously used the base-line survey data to investigate determinants and dimensions involved in self-rated health where a structural equation modelling was employed to test our theoretical model (Fylkesnes & Forde, 1992). The premise was that health and illness are defined normatively (Twaddle, 1974), such that subjective health evaluations are affected by cultural, socio-economic and structural frameworks assuming that roles and tasks represent an important frame of reference. Furthermore, the model suggested relations between health dimensions by employing measures of major chronic diseases and psychological distress together with physiological and behavioural measures as a construct of myocardial infarction risk score. The present analyses of the follow-up data are based on the same theoretical thinking.

Overall mortality was measured as deaths per 1000 person years of observation. Self-rated health was based on the response to the single item question: “How would you say your health is? Is it poor, fair (neither poor nor good), good, excellent?” The following sociodemographic variables were included as controls: age measured in years (30–62) and used both as a continuous variable and grouped (see more details under statistical analysis and Tables); sex (male/female); urban/rural place; civil status (coded married, single, divorced/separated/widow/-er); ethnic group (classification based on two questions: “Are two or more of your grandparents of Sami origin?” and “Are two or more of your grandparents of Finnish origin?” (coded Norwegian, Sami, Kven, Sami and Kven or ‘don't know’ and entered as dummies in regression analysis with Norwegian (majority group) as reference); receiving a full or partial work disability pension; and years of schooling, either used as years of completed schooling or grouped. Grouping had to differ by age because educational opportunities differed dramatically between the younger and the older group.

The following health status variables and physiological and health behaviour measures, were employed: chronic disease index (reported myocardial infarction, angina pectoris, diabetes, asthma, bronchitis, rheumatoid arthritis, cancer, migraine, epilepsy – coded to 0,2; being currently treated for high blood pressure; sleeplessness serious enough to hamper work ability (based on the questions “Are you bothered by sleeplessness?” – “Has sleeplessness been hampering your working abilities past year?” – coded to 0,2); high myocardial infarction risk (score based on serum cholesterol, systolic blood pressure, cigarettes currently smoked per day); triglyceride level (enzymatic method measured in mmol/l (1,4); BMI > 30 (0,1); and leisure physical activity (sedentary, moderate, keep-fit exercise, athletes).

### 2.3 Statistical analysis

The endpoint dependent variable in survival analyses was the survival time (years) and the survival status at the end of follow-up (survival time from the interview in 1987/88 to the date of death). The censored survival time of individuals alive at the end of the follow-up was calculated as the number of years from the interview to the end of 2017. The average censored survival time in the sample was 25.8 years, and 62.1% were still alive at the end of follow-up. The effect on all-cause mortality was estimated by the Cox proportional hazard models with hazard ratio (HR) and 95% confidence interval (CI) used as effect size. First, overall mortality and age-adjusted HR were estimated separately for men and women and for the age-groups 30–49 and 50–62 years. This stratification was employed for major estimates and the rationale was assumptions about relevant sex and age differences and identified interactions in the material (SRH*age, p = 0.048 – and SRH*sex, p = 0.000). Second, the predictive power of SRH for mortality was estimated by the stepwise inclusion of regression model variables. Third, the predictive power was analysed separately for educational groups. Different groupings for educational attainment was found to be necessary due to the substantially higher level of education in the youngest versus oldest group. Finally, we tested potential interactions by including interaction terms. The following supplementary analyses were performed with regards to a work disability pension and civil status: analyses of mortality risk and the predictive power of SRH for mortality risk.

## 3 Results

### 3.1 Person-years of observation and survival

**Fig. 1:**
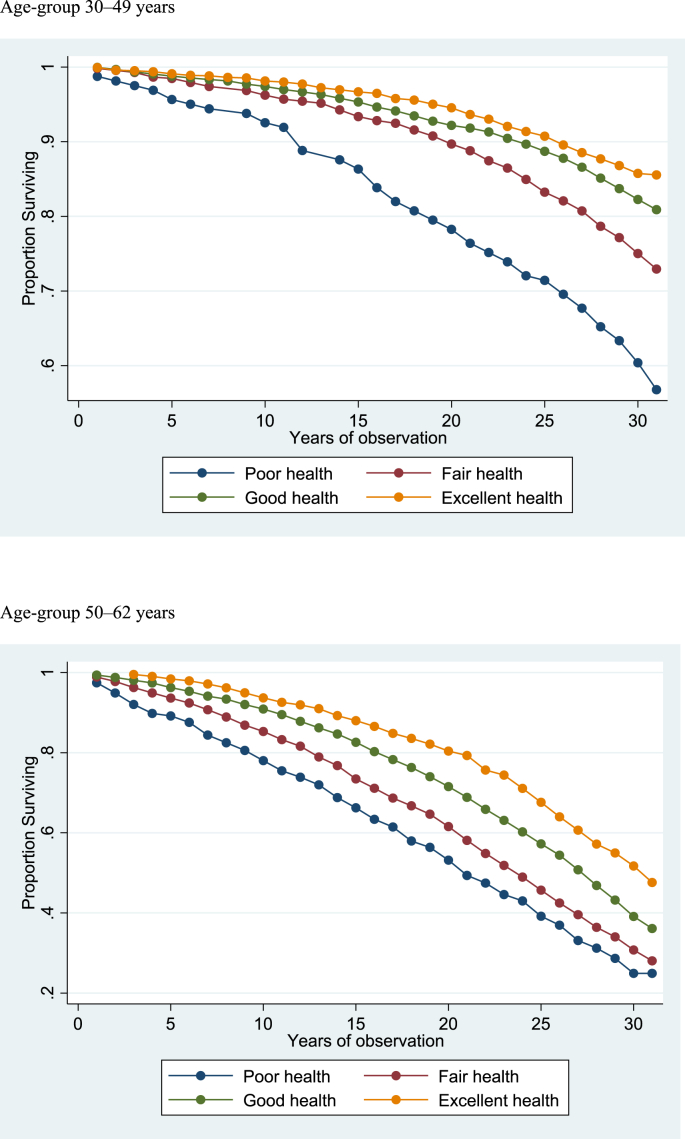
Life tables for survival for self-rated health, Finnmark cohort study 1987–2017.

With a mean of 25.77 years of observation at a total of 453,598 person-years, 6864 all-cause deaths were registered. The crude all-cause mortality rate for men was 18.36 (95% CI 17.80–18.94) and for women it was 12.07 (95% CI 11.62–12.53) per 1000 person-years. The age-adjusted mortality risk was 1.58 times higher in men compared with women (age-adjusted HR 1.58 (95% CI 1.50–1.66). There was a distinct difference in survival time over the entire follow-up period with self-rated health for both those aged 30–49 years and 50–62 years (Fig. 1).

### 3.2 Assessment of non-response bias

The possibility of distortion of mortality risk estimates due to non-response to the second and third questionnaires was analysed by comparing estimates based on participants in the examination versus respondents to the two final questionnaires (Table 1). These comparisons revealed only minor differences in the mortality estimates but with some loss in precision. Questions about SRH and years of schooling were both asked in the two final questionnaires and thus it is not possible to assess differential non-response. The variable work disability pension came from the first questionnaire and thus had only a marginal non-response. Further, since work disablement was strongly associated both with years of schooling and SRH, this variable could be indicative of differential non-response for education. The analyses showed only minor differences in the response comparing all responders versus only responders to questions about education and SRH.

**Table 1:** Differences in mortality rate between all participants in the screening and those who responded to the question on self-rated health (SRH), Finnmark cohort study 1987–2017.

	Participants N = 17,554 Deaths/1000 PYO^a^	Responders to questions on SRH only N = 12,383 (70.5%) Deaths/1000 PYO^a^
**Age-group**		
30–39	03.56 (03.16–04.02)	03.30 (02.84–03.82)
40–49	08.37 (07.98–08.78)	08.07 (07.62–08.56)
50–59	24.30 (23.49–25.50)	23.62 (22.70–24.58)
60–62	43.26 (41.09–45.51)	43.39 (40.98–45.92)
**Sex**		
Men	18.36 (17.80–18.94)	18.27 (17.62–18.95)
Women	12.07 (11.62–12.53)	12.17 (11.65–12.71)
**Work Disability Pension**		
No	12.68 (12.32–13.04)	12.56 (12.15–12.98)
Yes	30.90 (29.52–32.33)	31.39 (29.79–33.07)
**MIR**^b^		
No	13.84 (13.87–14.21)	13.84 (13.42–14.72)
Yes	26.34 (24.91–27.87)	26.77 (25.05–28.60)
**Cholesterol**		
Low	10.61 (10.01–11.16)	10.46 (09.86–11.09)
Middle	15.03 (14.43–15.66)	15.18 (14.47–15.92)
High	19.79 (19.09–20.53)	19.83 (19.01–20.70)

a PYO: Person years of observation.

b MIR: Myocardial infarction risk based levels of cholesterol, blood pressure and smoking.

### 3.3 Descriptive statistics

As shown in Table 2, reporting less than good health was substantially lower in the age-group 30–49 years (18.7%) compared with the older group (36.4%). The correlation (Pearson) between SRH and some of the variables in Table 2 were at the following levels: with educational attainment (-0.26), with work disablement (-0.37), with chronic disease index (-0.30) and with sleeplessness (-0.34). The correlation between work disability and educational attainment was −0.27. The distribution of years of schooling in the sample showed a marked difference in educational attainment between those aged 30–49 years and those 50–62 years (Table 2). In our analyses of differences between education groups, we are concerned with low versus high effects, and to end up with a reasonable number in education groups we therefore had to employ different groupings of education for the two age groups in the subsequent analyses. The supplementary analysis regarding the relationship between work disability pension and mortality risk revealed a substantial effect. In the age-group 30–49 years the age-adjusted Hazard Ratio was 2.26 for men and 2.25 for women. The respective effects for those aged 50–62 years were 1.71 and 1.51.

**Table 2:** Descriptive statistics of self-rated health, socio-demographic characteristics, health status, and risk measures, Finnmark cohort study 1987–2017.

	Age 30–49 years		Age 50–62 years	
	% in sample	% died during follow-up	% in sample	% died during follow-up
**Self-rated health**				
Poor/fair	18.7	27.3 (24.8–29.7)	36.4	70.4 (68.4–72.4)
Good	60.0	18.0 (16.8–19.2)	52.2	61.2 (59.4–63.0)
Very good	21.3	14.2 (12.4–16.0)	11.4	49.4 (46.0–53.4)
**Years of schooling**				
1–6 years	1.2	36.6 (25.9–47.2)	18.7	61.8 (58.7–64.9)
7 years	13.6	24.5 (21.7–27.3)	42.4	68.4 (66.4–70.3)
8–9 years	32.6	21.1 (19.3–22.8)	18.7	58.0 (54.8–61.1)
10–13 years	36.2	16.8 (15.3–18.3)	15.6	54.7 (51.2–58.1)
14+ years	16.4	12.8 (10.8–14.8)	4.7	55.8 (49.5–62.2)
**Work disability pension**				
No	93.8	18.7 (17.9–19.58)	70.4	9.5 (58.2–60.8)
Yes	6.2	37.8 (34.0–41.76)	29.6	74.5 (72.7–76.3)
**Civil status**				
Married	72.0	18.0 (17.1–18.9)	72.9	60.8 (59.5–62.0)
% Single	15.7	24.3 (22.1–26.4)	11.6	78.1 (75.4–80.8)
% Widowed/divorced	12.3	25.5 (23.0.27.9)	15.5	68.7 (66.0–71.2)
**High MIR-score**				
No	89.2	17.7 (16.9–18.5)	87.3	61.1 (60.0–62.3)
Yes	10.8	37.8 (34.9–40.8)	12.8	83.4 (81.1–85.8)
**Chronic disease index**				
0	76.7	18.4 (17.4–19.5)	66.7	60.4 (58.9–62.0)
1	19.9	19.3 (17.2–21.4)	23.7	65.6 (63.1–68.1)
>1	3.4	31.0 (25.0–37.0)	9.7	77.5 (74.0–81.0)
**Leisure physical activity**				
Sedentary	27.8	21.7 (20.1–23.2)	24.7	67.3 (65.2–69.4)
Moderate	55.8	19.9 (18.8–20.9)	63.7	63.0 (63.1–68.1)
Keep fit/athletes	16.4	16.7 (14.9–18.5)	11.7	61.8 (58.6–65.0)

**MIR-score: Myocardial infarction risk based levels of cholesterol, blood pressure and smoking.

### 3.4 Mortality prediction of SRH

SRH was a significant predictor of subsequent mortality for both sex and age-groups in all regression models (Table 3). Among those aged 30–49 years the age-adjusted predictive effect comparing poor health with excellent health as a reference was 2.90 (95% CI, 2.01–4.17) higher among men and 3.36 (95% CI, 2.16–5.24) higher among women. The corresponding estimates for the 50–62 years age-group were 2.21 (95% CI, 1.77–2.75) for men and 1.93 (95% CI, 1.47–2.52) for women. The strength of prediction markedly attenuated with the successive entry of predictors, particularly among the young women. Work disability pension appeared to have the most predominant confounding effect on prediction among both men and women and particularly in those aged 30–49 years. The adjustment effects of the various health status and risk-taking indicators were relatively modest.

**Table 3:** The predictive ability of self-rated health on mortality after 30 years of follow-up among men and women aged 30–62 years at baseline. Results from Cox proportional hazards model, Finnmark cohort study 1987–2017.

Self-rated health	Model 1: adjustments: Age (in years)	Model 2: adding civil status, urban place, ethnic group.	Model 3: adding work disability pension	Model 3: adding, years of schooling	Model 4: adding high myocardial infarction risk, triglycerid level, current treated with high BP, BMI>30	Model 5: adding Chronic diseases leisure physical activity, sleeping difficulties
	Hazard ratio (95% CI)	Hazard ratio (95% CI)	Hazard ratio (95% CI)	Hazard ratio (95% CI)	Hazard ratio (95% CI)	Hazard ratio (95% CI)
**Men** **Age 30–49**						
Poor	2.90 (2.01–4.17)	2.76 (1.92–3.99)	2.34 (1.59–3.44)	2.25 (1.51–2.38)	2.13 (1.40–3.22)	1.88 (1.21–2.92)
Fair	1.94 (1.56–2.42)	1.88 (1.50–2.34)	1.73 (1.37–2.17)	1.78 (1.29–1.90)	1.60 (1.25–2.05)	1.48 (1.14–1.92)
Good	1.22 (1.01–1.47)	1.20 (0.99–1.45)	1.18 (0.98–1.43)	1.22 (1.01–1.50)	1.18 (0.97–1.44)	1.15 (0.93–1.41)
Excellent	1	1	1	1	1	1
**Women** **Age 30–49**						
Poor	3.36 (2.16–5.24)	3.23 (2.07–5.05)	2.63 (1.66–4.17)	2.80 (1.75–4.48)	2.34 (1.45–3.80)	1.72 (1.04–2.88)
Fair	1.52 (1.11–2.07)	1.45 (1.06–1.98)	1.27 (0.92–1.75)	1.29 (0.92–1.80)	1.15 (0.81–1.62)	0.95 (0.66–1.37)
Good	1.28 (0.98–1.68)	1.25 (0.96–1.64)	1.23 (0.94–1.62)	1.26 (0.95–1.68)	1.22 (0.90–1.62)	1.09 (0.81–1.47)
Excellent	1	1	1	1	1	1
**Men** **Age 50–62**						
Poor	2.21 (1.77–2.75)	2.13 (1.70–2.66)	1.84 (1.46–2.31)	1.93 (1.52–2.46)	1.61 (1.25–2.06)	1.47 (1.13–1.92)
Fair	1.90 (1.62–2.23)	1.91 (1.62–2.24)	1.73 (1.47–2.04)	1.81 (1.51–2.16)	1.56 (1.30–1.88)	1.52 (1.25–1.85)
Good	1.28 (1.10–1.50)	1.31 (1.12–1.53)	1.29 (1.11–1.51)	1.34 (1.13–1.58)	1.28 (1.08–1.51)	1.26 (1.06–1.50)
Excellent	1	1	1	1	1	1
**Women** **Age 50–62**						
Poor	1.93 (1.47–2.52)	1.91 (1.46–2.51)	1.54 (1.16–2.04)	1.62 (1.20–2.20)	1.44 (1.06–1.96)	1.39 (1.00–1.94)
Fair	1.68 (1.37–2.05)	1.69 (1.38–2.07)	1.44 (1.17–1.78)	1.54 (1.23–1.93)	1.42 (1.13–1.78)	1.41 (1.10–1.80)
Good	1.43 (1.18–1.73)	1.44 (1.19–1.75)	1.36 (1.12–1.66)	1.42 (1.15–1.76)	1.34 (1.08–1.66)	1.32 (1.06–1.66)
Excellent	1	1	1	1	1	1

There was a significant interaction between SRH and years of schooling, i.e., SRH*years of schooling as interaction term, for poor/fair versus excellent/good health both for men (p = 0.019) and women (p = 0.028) in the age-group 50–62 years. The interaction was not statistically significant in age-group 30–39, although it was nearly significant for men (p = 0.073). The stratified analyses of mortality prediction of SRH by three education groups showed clearly overlapping confidence intervals (Table 4). However, for men aged 30–49 and men and women aged 50+, there was a consistent linear pattern of increasing mortality prediction of poor/fair health vs. good/excellent health (Table 4).

**Table 4:** Predictive power of self-rated health for mortality stratified by educational attainment groups, hazard ratios, and 95% confidence intervals, Finnmark cohort study 1987–2017.

	Men			Women		
	Age 30-49					
	Mortality for fair/poor SRH					
**Education**	Model 1	Model 2	Model 3	Model 1	Model 2	Model 3
**13** +	2.07 (1.30–3.27)	1.85 (1.15–2.96)	1.77 (1.06–2.95)	0.62 (0.23–1.74)	0.51 (0.18–1.46)	0.36 (0.11–1.13)
**9–12**	1.64 (1.27–2.11)	1.56 (1.20–2.03)	1.41 (1.05–1.88)	1.43 (1.04–1.96)	1.25 (0.89–1.75)	1.41 (1.05–1.88)
< **9**	1.68 (1.34–2.15)	1.47 (1.15–1.89)	1.26 (0.93–1.23)	1.49 (1.10–2.02)	1.31 (0.95–1.80)	1.23 (0.93–1.63)
	**Age 50**–**62**					
	**Mortality for fair/poor SRH**					
**Education**	Model 1	Model 2	Model 3	Model 1	Model 2	Model 3
**10** +	2.00 (1.57–2.56	1.79 (1.37–2.33)	1.54 (1.14–2.07)	1.61 (1.20–2.17)	1.40 (1.00–1.98)	1.46 (1.00–2.12)
**8–9**	1.49 (1.18–1.88)	1.37 (1.07–1.76)	1.32 (1.00–1.76)	1.40 (1.08–1.82)	1.18 (0.87–1.61)	1.23 (0.87–1.72)
**1–7**	1.48 (1.32–1.67)	1.33 (1.18–1.51)	1.16 (1.01–1.34)	1.17 (1.03–1.33)	1.06 (0.92–1.21)	0.97 (0.83–1.14)

Education: recoded from years of schooling; Model 1: adjustment age in years; Model 2: adding civic status as dummy variables (singe vs. widowed/divorced with married as control) and work disability pension; Model 3: adding myocardial infarction risk, treated for high blood pressure (0,1), triglyceride level, body mass index 30+, chronic diseases, sleeplessness, physical activity.

### 3.5 Education inequalities in mortality

Age-adjusted education inequalities in mortality appeared irrespective of age-group and sex but was clearly less pronounced in the older compared with the younger age-group (Table 5). Using different groupings of education for the two age-groups was found to be valid based on the results from analysing mortality using years of schooling as a continuous variable. Women aged 30–49 years with the lowest level of education had a 68% higher mortality than the most educatied group, and the increase in mortality was linear in shape. The effect was also substantial for men in this age-group, but it tended to be concave (down) due to a slight drop in the relative inequality in the group with the lowest education. A shape similar to that of the younger men also appeared for both sexes in the older age-group. The change in the inequalities with the stepwise adjustment in the age-group 30–49 was a successive narrowing of the gap in general. The major effect was seen in the group with the least education leading to a non-significant difference in the hazard ratio when comparing the highest and lowest education groups. The key modifying factor involved was work disability pension which had a strong correlation with education (Table 5). Single civil status was found with a substantial excess mortality (overall mortality risk 1.78, 95% CI, 1.67–1.91) but as it is modestly correlated with education, this factor did not substantially affected inequality. Finally, for those aged 50–62 years, the relatively smaller education inequality was not statistically significant after the stepwise adjustment.

**Table 5:** Educational inequalities in mortality risk. Results from Cox proportional hazards model, Finnmark cohort study 1987–2017.

Level of education	Model 1: adjustments: age in years	Model 2: adding civil status^a^	Model 3: adding work disability pension	Model 4: adding health status and risk
	Hazard ratio (95% CI)	Hazard ratio (95% CI)	Hazard ratio (95% CI)	Hazard ratio (95% CI)
Age 30–49 Men				
14+	1	1	1	1
10–13	1.31 (1.03–1.65)	1.28 (1.01–1.62)	1.26 (1.00–1.60)	1.18 (0.93–1.50)
8–9	1.66 (1.31–2.09)	1.59 (1.26–2.01)	1.54 (1.22–184)	1.39 (1.10–1.77)
1–7	1.40 (1.08–1.82)	1.28 (0.99–1.66)	1.17 (0.90–1.53)	1.00 (0.76–1.31)
**Women**				
14+	1	1	1	1
10–13	1.44 (1.02–2.02)	1.45 (1.03–2.03)	1.40 (1.00–1.97)	1.32 (0.93–1.85)
8–9	1.56 (1.11–2.17)	1.56 (1.11–2.18)	1.46 (1.05–2.06)	1.39 (0.99–1.95)
1–7	1.68 (1.16–2.44)	1.65 (1.14–2.39)	1.44 (0.98–2.10)	1.20 (0.81–1.78)
**Age 50–62 Men**				
10+	1	1	1	1
8–9	1.11 (1.34–1.29)	1.08 (0.93–1.26)	1.03 (0.88–1.19)	0.97 (0.82–1.12)
7	1.23 (1.08–1.39)	1.03 (1.03–1.33)	1.09 (0.96–1.24)	1.06 (0.93–1.21)
1–6	1.22 (1.06–1.41)	1.15 (0.99–1.14)	1.05 (0.91–1.22)	1.00 (0.86–117)
**Women**				
10+	1	1	1	1
8–9	1.07 (1.28–2.28)	1.07 (0.88–1.29)	1.03 (0.86–1.24)	1.00 (0.82–1.22)
7	1.36 (1.79–2.96)	1.36 (1.16–1.59)	1.27 (1.09–1.49)	1.24 (1.05–1.46)
1–6	1.22 (1.02–1.49)	1.22 (1.11–1.48)	1.11 (0.92–1.35)	1.07 (0.88–1.31)

a Civil status: single vs. divorced/widowed and married as reference; health status and risk: myocardial infarction risk, treated for high blood pressure (0,1), triglyceride level, body mass index 30 > 30, chronic diseases, sleeplessness, physical activity.

### 3.6 Education inequalities in SRH

The age-adjusted educational inequalities in self-rated health were substantially wider than for mortality risk across groups (Table 6). Both age-groups and sex inequalities were closely similar in magnitude with a linear shape. Interestingly, the particularly strong effect pattern of work disability on the least educated group was similar to the educational inequalities in mortality. This adjustment effect also appeared when estimating the predictive power of SRH on mortality (as shown in Table 3).

**Table 6:** Educational inequities in poor/fair SRH. Results from generalised linear model reporting rate ratios, Finnmark cohort study 1987–2017.

Level of education	Model 1: adjustments: age in years	Model 2: adding civil status^a^	Model 3: adding work disability pension	Model 4: adding health status and risk
	Rate Ratio (95% CI)	Rate Ratio (95% CI)	Rate Ratio (95% CI)	Rate Ratio (95% CI)
**Age 30–49 Men**				
14+	1	1	1	1
10–13	1.42 (1.06–1.91)	1.41 (1.05–1.89)	1.35 (1.00–1.81)	1.23 (0.92–1.67)
8–9	2.15 (1.62–2.87)	2.10 (1.58–2.80)	1.90 (1.42–2.54)	1.77 (1.32–2.38)
1–7	2.38 (1.75–3.27)	2.29 (1.67–3.13)	1.87 (1.36–2.58)	1.68 (1.21–2.34)
**Women**				
14+	1	1	1	1
10–13	1.38 (1.00–1.90)	1.37 (1.00–1.89)	1.30 (0.94–1.79)	1.24 (0.90–1.72)
8–9	2.29 (1.69–3.12)	2.28 (1.68–3.10)	2.04 (1.50–1.78)	1.98 (1.45–2.71)
1–7	2.92 (2.09–4.08)	2.84 (2.03–3.97)	2.19 (1.56–3.09)	1.98 (1.40–2.80)
**Age 50–62 Men**				
10+	1	1	1	1
8–9	1.57 (1.23–2.03)	1.56 (1.21–2.01)	1.40 (1.08–1.80)	1.37 (1.05–1.78)
7	1.89 (1.52–2.35)	1.84 (1.48–2.29)	1.53 (1.22–1.92)	1.61 (1.28–2.02)
1–6	2.44 (1.94–3.06)	2.36 (1.87–2.97)	1.89 (1.50–2.39)	1.78 (1.39–2.27)
**Women**				
10+	1	1	1	1
8–9	1.47 (1.15–1.87)	1.47 (1.15–1.87)	1.33 (1.04–1.70)	1.31 (1.01–1.68)
7	1.76 (1.43–2.18)	1.76 (1.43–2.18)	1.44 (1.17–1.79)	1.42 (1.14–1.77)
1–6	2.23 (1.78–2.82)	2.23 (1.77–2.82)	1.72 (1.36–2.17)	1.62 (1.27–2.07)

a Civil status: single vs. divorced/widowed and married as reference; health status and risk: myocardial infarction risk, treated for high blood pressure (0,1), high triglyceride level, body mass index >30, chronic diseases, sleeplessness, physical activity.

## 4 Discussion

Self-rated health is frequently used in health equity research due to its simplicity and unique ability to predict subsequent mortality. The predictive ability seems to differ with contextual factors, however, and so there must be careful validation efforts. We found the overall predictive power of self-rated health to be remarkably strong in this long-term follow-up. The predictive power tended to increase with educational attainment in those aged 50–62 years but not the younger group. Education inequality was considerable for both mortality and SRH, but with some marked differences. The education inequality in SRH was consistently wider and more linear in relationships across age and sex. The corresponding mortality gap clearly narrowed with age, which might partly be explained by selective mortality and historical changes in health equities. Moreover, the correspondence of the two measures/outcomes of educational inequality was underscored by the consistency in mutual predictors. We conclude that the results have relevance and important policy implications.

### 4.1 Mortality prediction of SRH

These finding agrees with previous research that shows that self-rated health is a strong predictor of subsequent mortality even after controlling for socio-demographics, work disability pension, and various health status dimensions including chronic diseases, psychological, behavioural and physiological measures (DeSalvo et al., 2006; Jylha, 2009; Lorem et al., 2020). Given a follow-up period of 30 years, SRH was a remarkably strong predictor of subsequent mortality. SRH as measured at the base-line study is likely to be somewhat misclassified compared to what would have been found if it had been measured at regular intervals during the follow-up period due to dilution of the relative risk estimates. Previous studies have found the predictive ability to weaken with the time of follow-up (Jylha, 2009). A recent study from Norway showed that the age and sex–adjusted hazard ratio for mortality (contrasting poor and very good health) dropped from 4.7 to 2.1 when comparing the first five years of follow-up to 15–21 years of follow-up (Lorem et al., 2020). A different but related dimension is about the impact of ongoing historical changes in the mortality prediction of SRH. There have been speculations that SRH's power of prediction might have gradually deteriorated with time along with new political ideologies such as healthism and the paradox of health. Healthism is a form of medicalisation characterised by a growing fascination with personal health, victim blaming, and lowering of the threshold of uneasiness or disease, pain, and help-seeking (Barsky, 1988; Buffel et al., 2017; Crawford, 1980). In contrast, a more recent study suggests that the predictive ability of SRH has increased over time (Schnittker & Bacak, 2014). The authors suggest that individuals may include more objective information in their assessments, such as mortality-related conditions, than they did in the past (Schnittker & Bacak, 2014). Although these results are based on limited observations over a short period of time, they are highly exciting to follow up for future validation studies of SRH.

### 4.2 Educational differentials in mortality prediction

Relatively few studies have explored whether the mortality prediction of SRH differs by socioeconomic position, but these few disclose interesting differences between countries. Studies from the US consistently show the prediction to increase with socioeconomic position (Dowd & Zajacova, 2007; Huisman et al., 2007; Schnittker & Bacak, 2014). Most studies from Europe, however, have shown insignificant or minor differences (Burstrom & Fredlund, 2001; Dalen et al., 2012; Holseter et al., 2015; McFadden et al., 2009; van Doorslaer & Gerdtham, 2003). In Netherlands, the predictive ability was found to increase with education in men but not in women (Huisman et al., 2007). The only study demonstrating the prediction ability to weaken was conducted in France (Singh-Manoux et al., 2007), contrasting the results from Spain that showed an increase in predictive ability by educational attainment (Regidor et al., 2010). The above pattern of different results by country has been suggested to be related to structural inequality (McFadden et al., 2009). Wide predictive gaps were mostly found in countries with the greatest inequalities (economically, in access to health care, and in educational opportunities). We found the ability of mortality prediction of SRH to increase with educational attainment only in men and women aged 50–62 years. This interaction was not significant in the younger age-group, although it was borderline significant for the men

In that age-group. Inequality regarding access to education/health care seems an unlikely explanation for this divergent finding. Norwegian regions differ in many ways but are relatively similar with regards to universal access to health care and education. A potential explanation might be the history of relatively high prevalence of work disability pensions among the least educated in those aged 50+ years, which is partly related to the resource crisis in the commercial fisheries during a period before and after the base-line study.

### 4.3 Inequality in mortality

A register-based examination of Norwegian residents over 34 years during the period 1961–2009 showed a steep increase in relative inequalities in life expectancy by educational attainment in both sexes (Steingrimsdottir et al., 2012). This rising trend in inequality took place during five decades of an overall upward trend in life expectancy. The widening of the education gap was explained by differential gains in life expectancy with educational attainment (Steingrimsdottir et al., 2012). Absolute inequalities in mortality stabilised during 2000–2010 for men due to reduced inequalities in cardiovascular mortality, whereas it continued to widen among women mostly due to lung cancer and chronic lung disease (Strand et al., 2014). A study in six countries in Europe revealed similar rising trends in relative inequalities in all-cause mortality in the period 1970–2010, and that cardiovascular diseases had lost their role as the primary contributor to these inequalities (de Gelder et al., 2017).

The education inequality in relative mortality was clearly highest in the younger group.

The validity of employing a different grouping of education in the youngest versus the older age-group can be questioned. The grouping was assessed by estimating mortality per year change in schooling, and these estimates supported the pattern of education inequality. The substantial age contrast in education inequality might partly be explained by selective mortality caused by the social gradient in mortality (i.e., the disadvantaged die at a younger age), thus reducing the gap in mortality at later ages (Benzeval et al., 2011; Huisman et al., 2007). The slightly concave mortality gradient seen for both older men and women in our data is an indication of this trend, since selective mortality is likely to be more pronounced among the very least educated. The age difference in the educational mortality gap could also be explained by historical changes in the gradient. A recent comprehensive examination of social class inequality in mortality in Sweden covered the period 1813 to 2015 revealed that a mortality gradient first emerged in the second half of the twentieth century (Bengtsson et al., 2020). Given the local relevance of this transition, the timing seems to match with the different lifecycle of our two cohorts.

### 4.4 Excess mortality of work disability pension

Our finding of the high excess mortality related to having work disability pension is consistent with previous studies conducted in Scandinavia. These studies found that adjusting for measures of health status explained only a fraction of the excess mortality (Gjesdal et al., 2008; Quaade et al., 2002; Wallman et al., 2006). Furthermore, that the excess mortality was not explained by the reason for the work disability pens, nor was that reason for the disability pension associated with cause of death (Wallman et al., 2006). This pattern indicates that cause of high excess mortality is complex and that factors other than those related to the disability diagnosis per se are important. Previous research has also consistently shown a high excess mortality linked with of unemployment, an effect also shown to be skewed towards low socioeconomic position groups (Clemens et al., 2015; Vagero & Garcy, 2016). Factors at work seem to have devastating effects on the life situation of people experiencing exclusion from work opportunities (Schuring et al., 2007), which is in line with the theory of the fundamental role that work plays in the life of men.

### 4.5 Medicalisation of unemployment

The local context at the time of the base-line survey was that this was a period of high unemployment and increasing numbers of work disability pensions (Nødtvedt, 1985). During the subsequent 30 years, there has been a general trend of increasing numbers of individuals on work disability pensions in the Scandinavian countries. In Norway the steepest increase during recent decades has been among young adults (Sveinsdottir et al., 2020). Epidemiological research on work disability pension in the Nordic countries have mostly focussed upon individual risk factors, and have come to the conclusion that these individual factors alone cannot explain the last decade's growth in work disability (Bjørngaard JH, 2009) and that measures of health status cannot explain the striking educational difference in receiving work disability pension (Ostby et al., 2011). Explanations of this escalation are mainly structural including verified political decisions intended to mask unemployment (Kolberg, 1972). Accordingly, a core structural problem has been converted to a health problem, an example of medicalisation, which can be considered a paradox of the welfare state (Buffel et al., 2017; Crawford, 1980). The steep social gradient in granting pensions also illustrates that social marginalisation is an unintended result of medicalisation (Buffel et al., 2017). These findings have important policy implications. For instance, efforts to reduce marginalisation imposed by preventable workforce exclusions could be part of a strategy to ameliorate massive loss of opportunities for individuals and society, and the resulting increase in health inequities.

### 4.6 Strengths and limitations

All residents in the Finnmark region aged 40–62 years were invited to participate in the base-line study, and two particular strengths of the study are the high attendance (87% turned up for the examination) and the 30 years of follow-up of mortality data (dates and causes of death identified by linkage to the Norwegian Cause of Death Registry, which was also crucial for the study). Additional data were collected after the examination using two self-administered questionnaires that were supposed to be filled in and returned by surface mail. The non-response to these questionnaires was substantial, which means there is a potential for a serious distortion of estimates. Fortunately, the high attendance in the examination offered an opportunity to assess the magnitude of non-response bias by comparing mortality estimates based on either including or excluding non-responders, respectively. Overall, these comparisons showed that the loss in precision of mortality estimates was insignificant.

Another concern relates to an extraordinarily high mobility in the region at the time of the base-line survey and for several years of the follow-up period. This mobility was explained by the sudden resource crisis in the fisheries causing great deal of uncertainties in terms of future employment opportunities (this crash is also thought to have caused high dependence on the work disability pension). Our data lack mobility information at the individual level, which makes geographical comparisons in mortality rather difficult to perform or interpret.

In conclusion, the power of SRH to predict mortality was exceptional after 30 years of follow-up. The magnitude of predictive power tended to increase with educational attainment in those aged 50 and over. SRH seems to add unique information essential to our understanding of health inequities. In addition to predicting the fatal effects of social selection mechanisms, SRH adds non-fatal effects and seems less prone to selective mortality. Educational inequality in mortality was substantial in the age-group 30–49 years but narrowed noticeably with age. Selective mortality may be part of the explanation together with historical changes in health equities. Work disability pensions appeared as the common key factor affecting educational inequality through strong effects on both SRH and subsequent mortality. The results are clearly relevant for methodological consideration in health equity research and have important policy implications. For instance, strengthen strategies to reduce marginalisation imposed by preventable workforce exclusions.

## Funding

This research received a grant from the Regional Research Fund, Region Northern-Norway (Regionalt forskningsfond - fondsregion Nord-Norge). No funding was received from other public, commercial, or not-for-profit actors.

## Ethical statement

Data were collected by the National Health Screening Services of Norway. The Norwegian Institute of Public Health was instrumental in providing the data and by arranging the linkage to the Norwegian Cause of Death Registry. The study was approved by the Regional Committee for Medical and Health Research Ethics (REK-Nord).

## CRediT authorship contribution statement

**Knut Fylkesnes:** Conceptualization, Data curation, Methodology, Formal analysis, Writing – original draft, Visualization. **Monika Dybdahl Jakobsen:** Writing – review & editing. **Nils Oddbjørn Henriksen:** Conceptualization, Project administration, Funding acquisition, Writing – review & editing.

## Declaration of competing interest

The authors declare that they have no conflict of interest.
